# Supplementary material for: Modeling glioblastoma heterogeneity as a dynamic network of cell states
Source: Mol Syst Biol. 2021 Sep 16;17(9):e10105. doi: 10.15252/msb.202010105 (PMC8444284; doi:10.15252/msb.202010105)
Supplement: Supplementary file 5 — Source Data for Figure 3 [file MSB-17-e10105-s001.zip › Figure3A_sourcedata/GSEA_3065/nosigdb_state3.GseaPreranked.1622718426028/NB_KI_CLUSTER4.html]

Details for gene set NB\_KI\_CLUSTER4[GSEA]

|  || Dataset | state3 |
| Phenotype | NoPhenotypeAvailable |
| Upregulated in class | na\_neg |
| GeneSet | NB\_KI\_CLUSTER4 |
| Enrichment Score (ES) | -0.49597842 |
| Normalized Enrichment Score (NES) | -1.7408339 |
| Nominal p-value | 0.0 |
| FDR q-value | 0.00888759 |
| FWER p-Value | 0.13 |
Table: GSEA Results Summary

  

Fig 1: Enrichment plot: NB\_KI\_CLUSTER4      
 Profile of the Running ES Score & Positions of GeneSet Members on the Rank Ordered List

  

| PROBE | GENE SYMBOL | GENE\_TITLE | RANK IN GENE LIST | RANK METRIC SCORE | RUNNING ES | CORE ENRICHMENT || 1 | HSP90AB1 |  |  | 74 | 0.371 | 0.0162 | No |
| 2 | SYT11 |  |  | 111 | 0.335 | 0.0344 | No |
| 3 | SEC11C |  |  | 179 | 0.295 | 0.0464 | No |
| 4 | ZNF704 |  |  | 188 | 0.291 | 0.0649 | No |
| 5 | MEG3 |  |  | 258 | 0.260 | 0.0743 | No |
| 6 | LUC7L3 |  |  | 289 | 0.247 | 0.0873 | No |
| 7 | SLC38A1 |  |  | 459 | 0.207 | 0.0816 | No |
| 8 | PHF14 |  |  | 504 | 0.198 | 0.0898 | No |
| 9 | ATP6V0A1 |  |  | 562 | 0.191 | 0.0959 | No |
| 10 | MTSS1 |  |  | 576 | 0.189 | 0.1070 | No |
| 11 | RUFY3 |  |  | 848 | 0.153 | 0.0859 | No |
| 12 | TUBB2B |  |  | 904 | 0.148 | 0.0895 | No |
| 13 | ZC3H13 |  |  | 908 | 0.148 | 0.0990 | No |
| 14 | MAP1B |  |  | 975 | 0.140 | 0.1007 | No |
| 15 | PEG10 |  |  | 987 | 0.139 | 0.1088 | No |
| 16 | ATP1B1 |  |  | 1102 | 0.129 | 0.1042 | No |
| 17 | CXADR |  |  | 1126 | 0.128 | 0.1101 | No |
| 18 | ATCAY |  |  | 1327 | 0.113 | 0.0946 | No |
| 19 | NSD3 |  |  | 1497 | 0.103 | 0.0820 | No |
| 20 | STMN3 |  |  | 1523 | 0.102 | 0.0858 | No |
| 21 | KIF3A |  |  | 1645 | 0.095 | 0.0782 | No |
| 22 | CADM1 |  |  | 1735 | 0.090 | 0.0739 | No |
| 23 | TSPAN7 |  |  | 1738 | 0.089 | 0.0797 | No |
| 24 | RIC3 |  |  | 1804 | 0.087 | 0.0779 | No |
| 25 | KIF21A |  |  | 1855 | 0.084 | 0.0777 | No |
| 26 | MIAT |  |  | 2016 | 0.075 | 0.0643 | No |
| 27 | MYEF2 |  |  | 2269 | 0.065 | 0.0396 | No |
| 28 | NELFCD |  |  | 2426 | 0.060 | 0.0255 | No |
| 29 | GDAP1 |  |  | 3303 | 0.033 | -0.0734 | No |
| 30 | APLP1 |  |  | 3403 | 0.030 | -0.0828 | No |
| 31 | TSPAN13 |  |  | 3926 | 0.017 | -0.1419 | No |
| 32 | TUBB2A |  |  | 4022 | 0.015 | -0.1519 | No |
| 33 | GLCCI1 |  |  | 4180 | 0.010 | -0.1693 | No |
| 34 | GTF2I |  |  | 4264 | 0.008 | -0.1784 | No |
| 35 | TMEM14A |  |  | 4365 | 0.005 | -0.1896 | No |
| 36 | ENO2 |  |  | 4676 | -0.003 | -0.2252 | No |
| 37 | ICA1 |  |  | 5053 | -0.012 | -0.2678 | No |
| 38 | TCAF1 |  |  | 5116 | -0.014 | -0.2740 | No |
| 39 | VAMP2 |  |  | 5192 | -0.015 | -0.2816 | No |
| 40 | TRIM2 |  |  | 5974 | -0.039 | -0.3692 | No |
| 41 | MDK |  |  | 6242 | -0.048 | -0.3968 | No |
| 42 | ATAT1 |  |  | 6334 | -0.051 | -0.4039 | No |
| 43 | CFAP20 |  |  | 6406 | -0.053 | -0.4086 | No |
| 44 | FXYD6 |  |  | 6822 | -0.072 | -0.4517 | No |
| 45 | HDAC2 |  |  | 6905 | -0.076 | -0.4561 | No |
| 46 | PRKAR2B |  |  | 7104 | -0.088 | -0.4731 | No |
| 47 | STXBP1 |  |  | 7303 | -0.101 | -0.4893 | Yes |
| 48 | NREP |  |  | 7330 | -0.103 | -0.4854 | Yes |
| 49 | CENPV |  |  | 7393 | -0.108 | -0.4854 | Yes |
| 50 | CCND1 |  |  | 7456 | -0.113 | -0.4850 | Yes |
| 51 | CALM1 |  |  | 7459 | -0.113 | -0.4777 | Yes |
| 52 | QDPR |  |  | 7488 | -0.116 | -0.4732 | Yes |
| 53 | RBFOX2 |  |  | 7516 | -0.118 | -0.4684 | Yes |
| 54 | CDK6 |  |  | 7605 | -0.128 | -0.4700 | Yes |
| 55 | TERF2IP |  |  | 7622 | -0.129 | -0.4632 | Yes |
| 56 | YWHAE |  |  | 7628 | -0.131 | -0.4551 | Yes |
| 57 | CKB |  |  | 7659 | -0.134 | -0.4496 | Yes |
| 58 | CIRBP |  |  | 7753 | -0.146 | -0.4506 | Yes |
| 59 | STMN1 |  |  | 7808 | -0.153 | -0.4466 | Yes |
| 60 | NME1 |  |  | 7811 | -0.154 | -0.4366 | Yes |
| 61 | TUBB |  |  | 7813 | -0.154 | -0.4264 | Yes |
| 62 | MCRIP1 |  |  | 7872 | -0.162 | -0.4223 | Yes |
| 63 | OCIAD2 |  |  | 7906 | -0.167 | -0.4149 | Yes |
| 64 | CBX1 |  |  | 7995 | -0.184 | -0.4128 | Yes |
| 65 | C3orf14 |  |  | 8011 | -0.187 | -0.4020 | Yes |
| 66 | ATP6V0E2 |  |  | 8025 | -0.189 | -0.3909 | Yes |
| 67 | RBP1 |  |  | 8081 | -0.200 | -0.3839 | Yes |
| 68 | SOX4 |  |  | 8123 | -0.209 | -0.3747 | Yes |
| 69 | FSCN1 |  |  | 8144 | -0.214 | -0.3627 | Yes |
| 70 | GDI1 |  |  | 8161 | -0.218 | -0.3500 | Yes |
| 71 | NLRP1 |  |  | 8171 | -0.220 | -0.3363 | Yes |
| 72 | JPT1 |  |  | 8212 | -0.231 | -0.3255 | Yes |
| 73 | CALM2 |  |  | 8295 | -0.257 | -0.3178 | Yes |
| 74 | UCHL1 |  |  | 8299 | -0.258 | -0.3010 | Yes |
| 75 | PEBP1 |  |  | 8348 | -0.273 | -0.2883 | Yes |
| 76 | YWHAQ |  |  | 8360 | -0.278 | -0.2710 | Yes |
| 77 | YWHAH |  |  | 8384 | -0.289 | -0.2544 | Yes |
| 78 | DPYSL3 |  |  | 8391 | -0.291 | -0.2357 | Yes |
| 79 | MARCKSL1 |  |  | 8415 | -0.300 | -0.2183 | Yes |
| 80 | H3F3A |  |  | 8442 | -0.311 | -0.2005 | Yes |
| 81 | MLLT11 |  |  | 8489 | -0.335 | -0.1835 | Yes |
| 82 | TUBA1A |  |  | 8499 | -0.341 | -0.1618 | Yes |
| 83 | GNAS |  |  | 8512 | -0.344 | -0.1402 | Yes |
| 84 | SNRPN |  |  | 8543 | -0.360 | -0.1197 | Yes |
| 85 | SUMO2 |  |  | 8573 | -0.383 | -0.0974 | Yes |
| 86 | BEX3 |  |  | 8584 | -0.388 | -0.0727 | Yes |
| 87 | MYL6B |  |  | 8618 | -0.422 | -0.0484 | Yes |
| 88 | PTMS |  |  | 8642 | -0.447 | -0.0212 | Yes |
| 89 | H3F3B |  |  | 8687 | -0.504 | 0.0074 | Yes |
Table: GSEA details [plain text format]

  

Fig 2: NB\_KI\_CLUSTER4: Random ES distribution      
 Gene set null distribution of ES for **NB\_KI\_CLUSTER4**
